# Management of a First Branchial Cleft Fistula in an Adult Patient: A Case Report and Literature Review

**DOI:** 10.7759/cureus.87756

**Published:** 2025-07-11

**Authors:** Alexandros Louizakis, Asterios Antoniou, Dimitris Tatsis, Solon Politis, Anestis Chrisostomidis, Kalliopi Domvri, Ioannis Astreidis, Nicoleta Pastelli, Christos Giankoulof, Konstantinos Paraskevopoulos

**Affiliations:** 1 Department of Oral and Maxillofacial Surgery, Aristotle University of Thessaloniki, Thessaloniki, GRC; 2 Specialized Cancer Treatment and Reconstruction Centre, "George Papanikolaou" General Hospital of Thessaloniki, Thessaloniki, GRC; 3 Department of Histology and Embryology, Aristotle University of Thessaloniki, Thessaloniki, GRC; 4 Department of Pathology, "George Papanikolaou" General Hospital of Thessaloniki, Thessaloniki, GRC; 5 Department of Radiology, "George Papanikolaou" General Hospital of Thessaloniki, Thessaloniki, GRC

**Keywords:** branchial region, congenital abnormalities, facial nerve, fistula, parotid gland

## Abstract

First branchial cleft anomalies (FBCAs) are uncommon congenital malformations that develop due to incomplete closure of the first branchial cleft during embryonic development. They represent a small proportion of all branchial cleft anomalies. These lesions, which may present as cysts, sinuses, or fistulas, pose significant diagnostic and therapeutic challenges due to their nonspecific clinical manifestations and complex anatomical relationships, particularly with the facial nerve and parotid gland. This case report describes a 47-year-old male patient presenting with recurrent infections and purulent discharge in the right preauricular and submandibular regions, later diagnosed as a Work Type II first branchial cleft fistula extending from the external auditory canal (EAC) through the parotid gland to the submandibular skin. Diagnostic evaluation included high-resolution magnetic resonance imaging (MRI) and computed tomography (CT) fistulography, which confirmed the fistula’s course and its intimate relation to the facial nerve. Surgical management involved complete excision via a modified Blair incision, retrograde facial nerve dissection, and superficial parotidectomy, with intraoperative neuromonitoring to minimize nerve injury risk. Histopathology revealed a cutaneous fistulous tract lined with stratified squamous epithelium and sparse chronic inflammatory infiltrates. Postoperative recovery was uneventful, with transient House-Brackmann grade II facial nerve palsy resolving within four weeks and no recurrence at the 12-month follow-up. This case is notable for the patient’s unusually late presentation in adulthood, contrasting with the typical diagnosis in childhood. A comprehensive literature review highlights the embryological basis, clinical variability, and diagnostic challenges of FBCAs, emphasizing the critical role of preoperative imaging for delineating the fistula’s anatomy and planning surgery. Complete surgical excision with facial nerve preservation remains the gold standard, though the procedure is complicated by the lesion’s proximity to critical neurovascular structures. Intraoperative neuromonitoring and patient-specific surgical approaches are essential to minimize complications such as facial nerve palsy and recurrence. This case underscores the importance of timely diagnosis, meticulous preoperative planning, and specialized surgical expertise in achieving favorable outcomes for FBCAs, particularly in atypical adult presentations.

## Introduction

First branchial cleft anomalies (FBCAs) are rare congenital disorders arising from embryonic remnants of the first branchial cleft with an incidence of one in 100,000, accounting for 8%-10% of all branchial cleft anomalies [1,2]. These anomalies present significant challenges in diagnosis and treatment due to the non-specific clinical manifestations and complex anatomic relationships, especially with facial nerve branches [3,4]. FBCAs are characterized by a slight female predominance and a marginally increased incidence in the left cervical region, and they can occur as cysts (68%), sinuses (16%), or fistulas (16%) [5, 6].

During the fourth to seventh weeks of gestation, the ectodermal invagination between the first and second branchial arches forms the first branchial cleft. Under normal circumstances, this cleft is obliterated by the seventh week; however, failure of complete obliteration results in persistent epithelial-lined tracts or cystic formations [1,7]. These embryonic residues can extend from the submandibular region to the external auditory canal (EAC) and frequently traverse the parotid gland, in close relation to the facial nerve [8]. During surgical excision, the complex anatomy of the facial nerve and its proximity to the lesion significantly increase the risk of nerve injury [9].

Work’s classification system for FBCAs divides these lesions into two primary categories based on histology and anatomical course [10]. Type I lesions originate from the ectoderm and are typically located medial to the auricular concha and extend lateral to the facial nerve, usually presenting as cysts near the EAC. Type II lesions consist of both ectodermal and mesodermal elements, commonly extending through or lying deep within the parotid gland, and are often associated with sinuses or fistulas that traverse or are located between branches of the facial nerve [9,11].

Due to their rarity and nonspecific clinical manifestations, such as recurrent infections in the head and neck area, otorrhea, or swelling in the submandibular region, FBCAs are often misdiagnosed or subjected to multiple incisions and drainage procedures, which can cause fibrosis and complicate definitive treatment. [1,2]. Consequently, high-resolution magnetic resonance imaging (MRI) and computed tomography (CT) are necessary for a precise diagnosis and optimal surgical planning, allowing accurate delineation of the tract and its relationship with important adjacent neurovascular structures [12]. Surgical excision is the cornerstone treatment of these lesions, with intraoperative facial nerve monitoring advised to minimize the risk of facial nerve palsy and reduce the risk of recurrence [1].

The aim of this article is to present a rare case of an adult male patient with a Work Type II first branchial cleft fistula involving the external auditory canal and the parotid gland in an adult patient and to review the current literature.

## Case presentation

**Figure 1:**
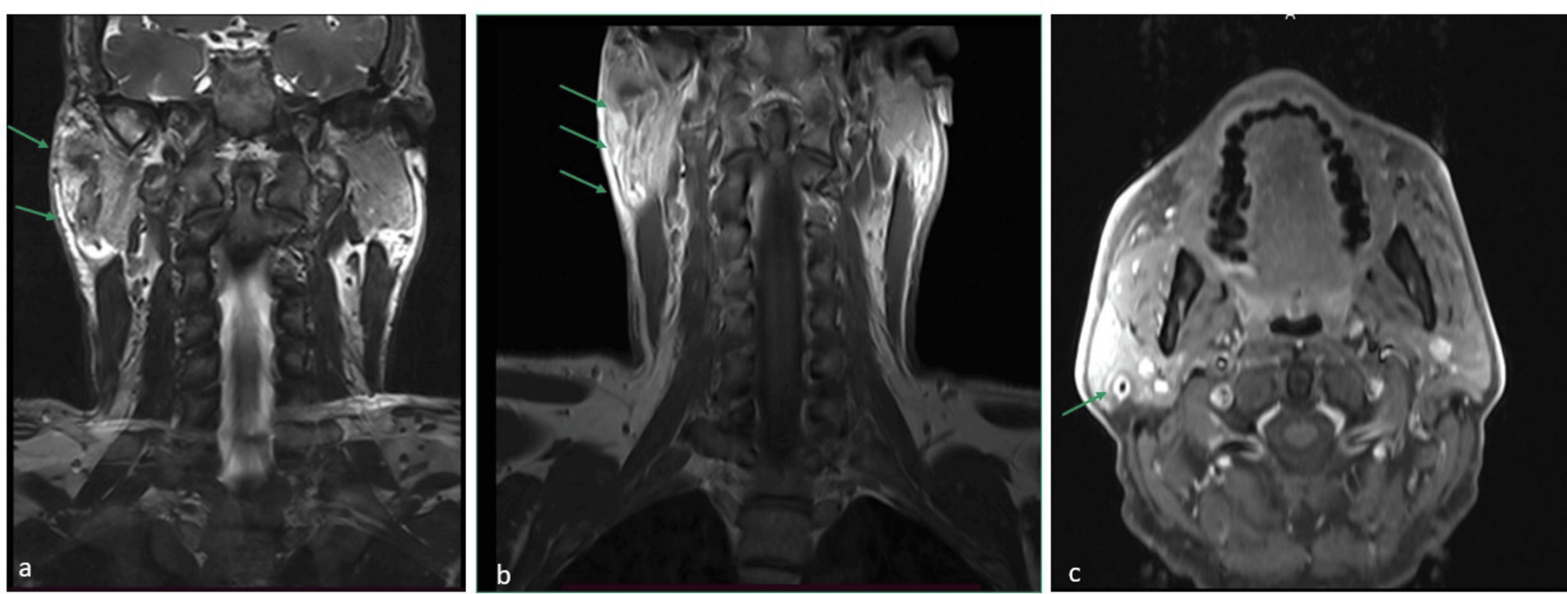
MRI showed an intraparotid lesion in the superficial layer, resembling a fistula (green arrows). The fistula tract varied in diameter, with the widest part measuring 1.6 cm in the middle. The external auditory canal and right tympanic membrane appeared normal, indicating no involvement of these structures. Nearby ear structures were not affected. (a) T2 sequence, coronal view; (b) T1 sequence, coronal view; (c) T1 sequence, axial view

A 47-year-old male patient visited the outpatient clinic for recurrent episodes of skin infections in the neck and the preauricular area on the right-hand side. The patient reported that these episodes had been present since his early childhood, but their frequency and severity had increased in the past few months, leading him to seek medical attention. His past medical history was otherwise unremarkable. Physical examination at the time of the first visit revealed a non-significant edema and a mild purulent discharge in the external auditory canal and the right submandibular region. Intraoral examination revealed no significant findings; thus, the probable cause of an odontogenic infection was excluded from the differential diagnosis. Laboratory exams did not reveal increased inflammation markers. To further investigate the underlying condition, an MRI was ordered. The MRI revealed an intraparotid lesion extending in the superficial layer, mimicking a fistula (Figure 1). The fistula tract was measured to have a variable diameter, with the greatest diameter situated in the mid portion, measuring 1.6 cm. Both the external auditory canal and the right tympanic membrane were found to be normal. The differential diagnosis of first branchial cleft fistula was set, with a low possibility of parotid tumor, lymphatic malformation, sialocele, or other infection. Due to the presence of skin tract-like lesions, a CT fistulography was deemed the next diagnostic step. 

**Figure 2:**
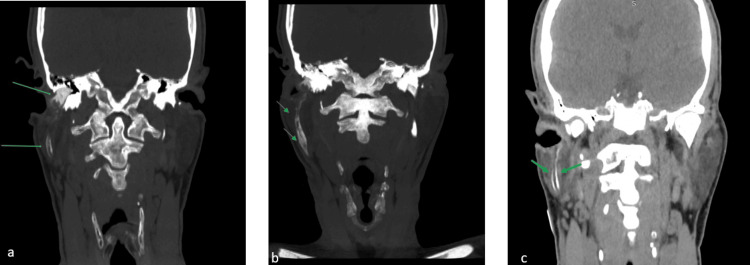
CT fistulography The green arrows depict a fistula with a maximum diameter of 1.6 cm, starting from the external auditory canal, continuing through the parotid tissue, and ending in the right submandibular skin region (a, b, and c show the coronal view).

**Figure 3:**
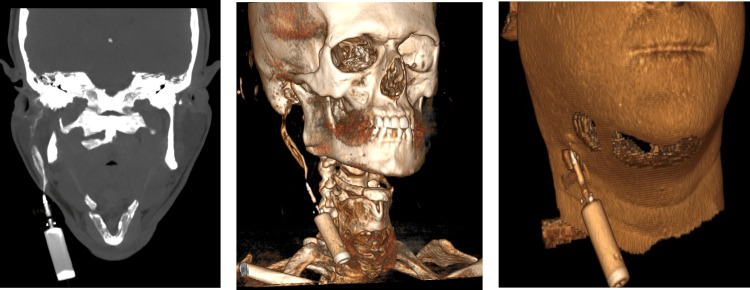
CT fistulography The green arrows depict the course of the fistula with a maximum diameter of 1.6 cm, starting from the external auditory canal, continuing through the parotid tissue, and ending in the right submandibular skin region (pane a shows the coronal view); (b and c) 3D rendering images show again the course of the fistula, demonstrating hard and soft tissue structures, respectively.

**Figure 4:**
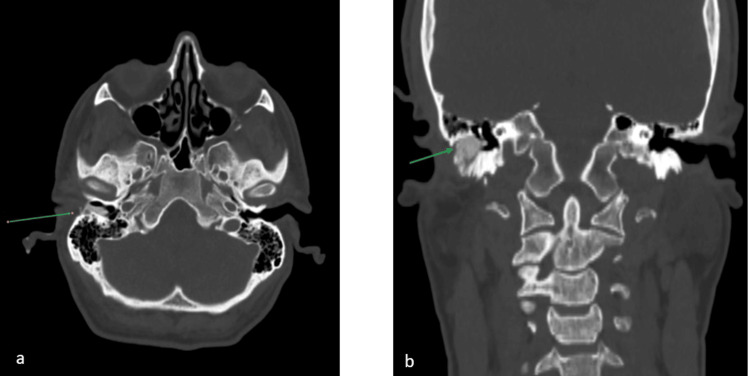
CT fistulography The green arrows reveal the fistula and its relation to the external auditory canal; (a) axial view, (b) coronal view.

**Figure 5:**
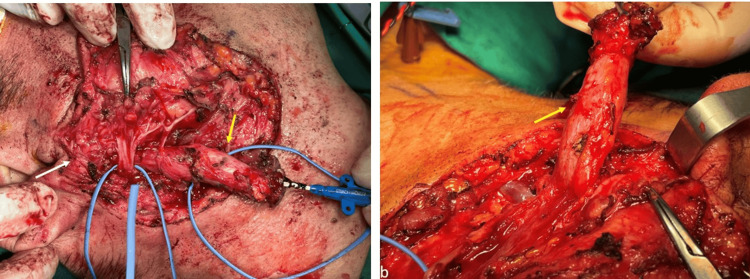
Clinical presentation of the branchial cleft fistula (a) The fistula passing through the parotid tissue with a course under the main trunk of the facial nerve; (b) The fistula after being dissected, with part of the parotid tissue after careful removal from the facial nerve. The yellow arrows depict the branchial cleft fistula.

**Figure 6:**
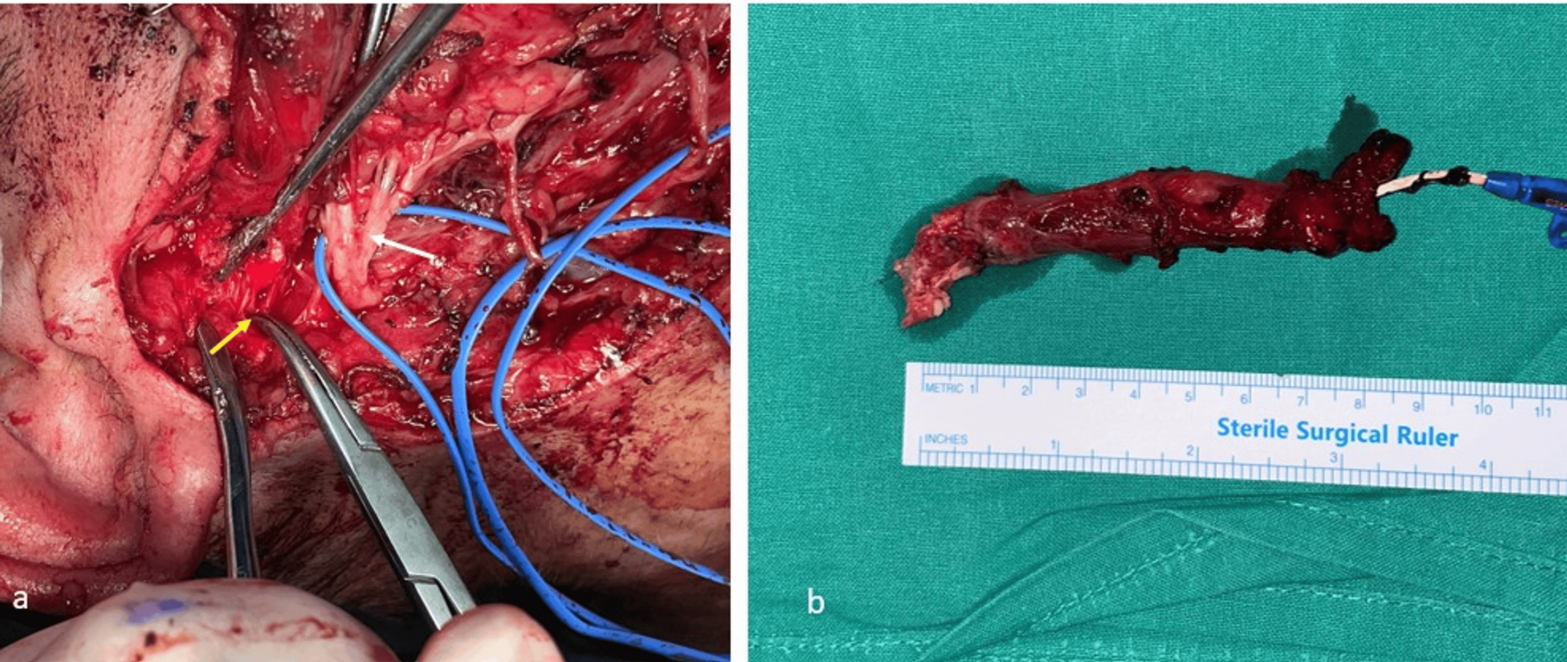
Clinical presentation of the branchial cleft fistula (a) The distal stroma of the fistula, situated in the anterior wall of the external auditory canal, was identified and completely removed (yellow arrow), facial nerve (white arrow); (b) The excised specimen, with a total length of 9 cm.

CT fistulography revealed a fistula that started from the EAC, continued through the parotid tissue, and ended in the submandibular skin region (Figures 2-4). Excision of the first branchial fistula was performed under general anesthesia. An initial peristomal incision in the submandibular area was designed, and a Blair incision was extended preauricularly. Facial nerve dissection was performed in a retrograde manner to preserve the marginal mandibular branch. The fistula tract was identified within the superficial lobe of the parotid gland, leading to the decision to perform a superficial parotidectomy (Figure 5). The distal stoma of the fistula was identified in the anterior wall of the external auditory canal and was completely removed (Figure 6). Closure was performed in a standard fashion, including suturing of the stoma edge in the external auditory canal. The postoperative period was uneventful, with mild dysfunction of the facial nerve (House-Brackmann score of 2), which resolved within four weeks.

**Figure 7:**
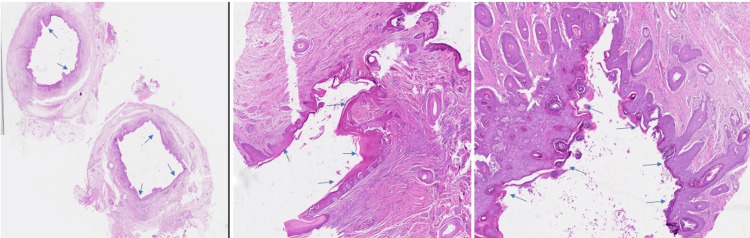
(a) Sinus tract covered by stratified squamous epithelium without atypia (hematoxylin and eosin, ×40); (b, c) fibrous connective tissue forming the wall of the sinus tract, without associated chronic inflammatory infiltrate or granulation tissue. Blue arrows depict the course of the track (hematoxylin and eosin, ×100).

The pathology report revealed a cutaneous fistula (fistulous tract). The tract was lined with a stratified squamous epithelium. Subepithelial sparse inflammatory infiltrates were not observed (Figure 7). 

## Discussion

FBCAs represent a rare subgroup of branchial apparatus malformations resulting from incomplete involution of the first branchial cleft during embryogenesis. They typically present as lesions with nonspecific clinical findings and close anatomical relationships with the facial nerve, which substantially increases the complexity of surgical management; thus, they pose a significant diagnostic and therapeutic challenge [1]. Embryologically, the external auditory canal and the lateral tympanic membrane are primarily derived from the first branchial cleft. Remnants of this area of the branchial apparatus can occur as cysts, sinuses, or fistulas extending from the EAC to the lateral side of the neck, often extending through or adjacent to the parotid gland [6, 13]. This close relationship with the parotid gland and facial nerve explains the high incidence of surgical complications and facial nerve injury reported in the literature [14].

Among the various systems proposed in the literature, Work’s classification system is the most used for dividing the FBCAs into two categories based on their histological features and embryological origin. Type I anomalies derive exclusively from ectoderm and commonly occur as cystic lesions situated near or medial to the concha, adjacent to the lateral side of the facial nerve. In contrast, Type II anomalies consist of both ectodermal and mesodermal elements and typically manifest as fistulas or sinuses. The fistulous tracts often traverse through or lie deep within the parotid gland, often with a close anatomical relationship with the branches of the facial nerve, complicating surgical management. [1,10]. Due to the presence of a fistulous tract extending from the EAC through the parotid gland and terminating at the skin of the submandibular area, our case aligns with the Work Type II classification.

Clinically, patients with FBCAs can present with a variety of symptoms extending from asymptomatic neck masses to recurrent infections, otorrhea, and purulent discharge from a sinus or fistula. These nonspecific clinical findings often lead to multiple ineffective treatments, such as incision and drainage or antibiotic therapy, before definitive surgical management [4, 7]. Our case, with a reported history of recurrent skin infections and purulent discharge in preauricular and submandibular regions and otologic symptoms, is consistent with prior literature reporting otologic involvement in up to 40% of FBCA cases [1, 2, 15].

CT and MRI findings in patients with FBCA lesions are necessary for differential diagnosis and preoperative surgical planning. High-resolution MRI provides enhanced soft tissue contrast, contributing to the identification of the extent of the fistulous tract and its relationship to the facial nerve and parotid gland [12]. CT fistulography complements MRI by delineating the tract’s relationship to adjacent bony structures and verifying its course from the EAC to the submandibular skin surface [12]. In our case, MRI revealed a fistulous tract traversing the parotid gland beneath the main trunk of the facial nerve, an unusual anatomical course that increases the intraoperative risk for facial nerve damage [12, 16]. CT fistulography confirmed these findings and provided additional anatomic details, which are crucial for preoperative surgical planning.

The gold standard in the treatment of FBCAs is the complete surgical excision of the fistulous tract with facial nerve preservation. The high risk of facial nerve injury, especially when the tract traverses deep into the parotid gland or surrounds branches of the facial nerve, makes the surgical approach challenging [17]. The use of neuromonitoring for early intraoperative identification of the facial nerve is recommended to minimize the risk of facial nerve palsy [2]. In our case, a retrograde facial nerve dissection and superficial parotidectomy through a modified Blair incision were performed to ensure complete excision of the fistulous tract. The unusual course of the fistulous tract under and in close relationship to the main trunk of the facial nerve, instead of a peripheral branch, made the procedure more challenging and required careful intraoperative management. Similar rare anatomical variants have been reported in the literature by Moriyama et al., highlighting the need for patient-specific surgical approaches based on intraoperative findings [4]. Delayed diagnosis or incomplete excision of FBCAs increases the risk of recurrent infections, fibrosis, and higher rates of facial nerve injury [9]. In cases with prior repeated drainage procedures, scar tissue formation distorts normal anatomical planes and complicates safe and effective surgical excision. Thus, early referral to specialized centers with experience in FBCAs is crucial [1, 17].

This case highlights the importance of an appropriate diagnosis and surgical plan. The difference in this case compared to most cases published in the literature is the fact that this patient was middle-aged, and this was not diagnosed during his childhood. Most branchial cleft anomalies are diagnosed and treated in childhood. The patient’s postoperative course was uneventful except for transient House-Brackmann grade II facial nerve palsy, which fully resolved within four weeks. No recurrence was observed at the 12-month follow-up, consistent with reports demonstrating that complete excision is the key factor in preventing recurrence [18, 19].

## Conclusions

FBCAs are rare congenital malformations that create significant diagnostic and surgical challenges due to their broad spectrum of clinical manifestations and close anatomical relationship with the facial nerve. Preoperative imaging with CT fistulography is essential for defining the tract and planning a safe surgical approach. Complete surgical excision with facial nerve preservation remains the gold standard in the treatment of FBCAs. Intraoperative neuromonitoring of the facial nerve and patient-specific surgical approaches are necessary to minimize complications. Our case highlights the importance of timely, carefully planned surgical intervention, which can result in successful outcomes.
